# ‘You just really have to assert yourself:’ social work, nursing, and rehabilitation counseling student experiences of providing integrated behavioral health services before and after the immediate start of COVID-19

**DOI:** 10.1186/s12913-022-07465-w

**Published:** 2022-01-18

**Authors:** Edward J. Alessi, Barbara Caldwell, Anthony S. Zazzarino, Brett Greenfield, Patricia A. Findley

**Affiliations:** 1 School of Social Work, Rutgers, The State University of New Jersey, 390 George Street, FL 6 – Room 607A, New Brunswick, NJ 08901 USA; 2 School of Nursing, Division of Advanced Nursing Practice, Rutgers, The State University of New Jersey, Newark, NJ USA; 3 School of Health Professions, Rutgers, The State University of New Jersey, Scotch Plains, NJ USA

**Keywords:** Primary care, Interprofessional education, Interprofessional collaborative practice, COVID-19, Healthcare students, Integrated behavioral health

## Abstract

**Background:**

Educators who train healthcare students to provide behavioral health services in primary care settings frequently encounter challenges as they work to ensure that students acquire the knowledge and skills to effectively function on interprofessional practice teams. This has become increasingly important during COVID-19, as interprofessional collaborative practice is needed more than ever to address the interrelated health, mental health, and social structural issues linked to the pandemic.

**Methods:**

We used qualitative focus groups to understand the experiences of 6 interprofessional teams (comprised of graduate social work, nursing, and rehabilitation counseling students; *n* = 19) providing behavioral health services in primary care settings before and after the immediate start of COVID-19. To triangulate data and enrich findings, one focus group with students’ faculty supervisors was also conducted; *n* = 5). Data were analyzed using thematic analysis.

**Results:**

Four themes highlighted student participants’ need to assert themselves at the beginning of their educational experience, to communicate and learn from one another to develop positive team dynamics, to contend with role confusion and missed opportunities for collaboration, and to manage the emotional impact of COVID-19 on learning.

**Conclusion:**

Findings indicate that educators should work with clinical faculty and agency supervisors to orient students to ensure they have role clarity within the agency. Graduate students providing behavioral health services should also learn to work collaboratively within their scopes of practice to serve patients virtually, especially in preparation for public health emergencies.

## Background

It has been well established that healthcare delivery in primary care is driven by complex interactions of medical, mental health, and social structural issues. Given this complexity, there is a recognized need to increase the level of support and services for individuals with psychiatric disorders, especially in primary care settings [1, 2]. It should be no surprise then that the integration of behavioral health in primary care delivered through interprofessional teams allows organizations to serve individuals with mental health issues by improving healthcare outcomes, increasing access, and reducing stigma and health disparities [3]. Educators who train students in social work, nursing, and rehabilitation counseling to provide behavioral health services frequently experience challenges, as they attempt to ensure that students in these disciplines acquire the knowledge and skills needed to work effectively in interprofessional practice teams [4].

Despite the importance of teamwork in the provision of integrated care [5], studies tend to examine interprofessional collaborative practice (ICP) using simulated scenarios or didactic case presentations [6, 7], instead of identifying the perspectives of graduate students actually working in interprofessional teams in integrated primary practice. Organizations also tend to vary in their level of integration [8] (i.e., some are fully integrated while others may only be just beginning the integration process), indicating that there is a strong need to understand how this impacts interprofessional learning as well as healthcare service delivery. Such an examination is especially warranted during the COVID-19 pandemic, as ICP has been considered essential for addressing the interrelated health, mental health, and social structural conditions linked to the pandemic and magnified by its protracted nature [9–11].

Thus, the purpose of this qualitative study was to describe and understand the experiences of graduate social work, nursing, and rehabilitation counseling students engaged in the delivery of behavioral health services in an integrated primary care setting. Our aim was to explore what their experiences were like prior to COVID-19 and what they were like in the few weeks after it began, in order to capture the nuances of engaging in ICP in real-life. Information from this study has the potential to fill a research gap in interprofessional care and education and improve behavioral health services provided in the primary care setting.

### Preparation for Interprofessional collaborative practice

As part of ensuring that graduate students in the behavioral health professions are effectively trained to provide services in primary care settings, the Interprofessional Educational Collaborative (IPEC) has developed a framework that is widely used to support students as they attempt to acquire the knowledge and skills necessary for ICP [12]. The IPEC framework is comprised of four competencies that provide the foundation for ICP: maintaining a team environment of mutual respect and shared values of patient-centered care; practicing at the highest level of one’s scope of practice; promoting effective team communication to ensure the best patient outcomes; and supporting overall team development for safe, timely, effective, and equitable team outcome [12]. This framework assists educators in helping healthcare students develop their professional identities, learn relevant knowledge, sharpen their skill set, identify their limitations, and understand what each discipline contributes to patient-care delivery (to optimize their scope of practice) [13, 14].

To help healthcare students meet the overarching learning objectives of their educational training in ICP, the social work, nursing, and rehabilitation counseling disciplines have acknowledged that students should be provided with both didactic and clinical experiences [15–18]. Using both modes of instruction provides the scaffolding for the implementation of interprofessional team-based care delivery [19], allowing teams to navigate the interaction of medical, psychiatric, and social structural problems through different lenses and perspectives [20]. Through a combination of didactic and clinical experiences, interprofessional teams from multiple disciplines influence one another in ways that enable interprofessional team members to recognize their own abilities and limitations [13]. This is vital since role confusion, divisions, and territoriality can still develop and persist in interprofessional teamwork. Thus, team members must make concerted efforts to alter their behaviors and engage in team collaboration in order to deliver optimal patient care [21]. To facilitate collaboration, team members must improve cooperation and communication and modify their thinking and behaviors [22]. Coaching has been considered one way to help team members do this, as it assists in facilitating discussion, building consensus, enhancing teamwork, and sharing information [23]. Another way to foster ICP among healthcare students is to have them engage in simulated practice experiences and subsequent debriefing sessions to identify their strengths and their limitations when providing team-based behavioral health services in primary care [23].

### Interprofessional collaborative practice and COVID-19

The need to provide interprofessional student teams with coaching and simulated practice experience is of paramount importance as the world continues to deal with the effects of the COVID-19 on both physical and mental health [24]. The prevalence of depression in the United States was approximately 3 times higher (27.8%) at the start of COVID-19 as compared to population-based estimates (8.5%) before COVID-19 [25]. Symptoms of posttraumatic stress disorder have also been reported in 3 out of 10 COVID-19 survivors and 2 out of 10 healthcare workers [26]. The pandemic has also exacerbated existing health disparities and precipitated a number of secondary psychosocial issues, including social isolation, financial insecurity, loss of employment, and scarcity of resources [11, 27].

Moreover, the provision of health services, including interprofessional interactions and collaboration, has changed because of the pandemic. In fact, Xyrichis and Williams have asserted that ICP “would be the only way health systems could respond successfully to the challenge of COVID-19” (p. 577) [28]. Studies that examine the impacts of COVID-19 on interprofessional practice have recently emerged. For example, a qualitative study of health and social care frontline workers in the United Kingdom showed they experienced numerous challenges at the start of COVID-19, including lack of pandemic preparedness, difficulties implementing social distancing protocols, shortage of personal protective equipment (PPE), delays in COVID-19 testing, and anxiety and fear among team members [29]. Findings from a case study, also conducted in the United Kingdom, revealed that bringing multiple disciplines (nursing and allied health) at a cardiothoracic hospital together to work in one team ensured smooth collaboration and communication when engaging in rapid responses to the pandemic [30]. This collaboration was facilitated by co-location (i.e., situated in the same practice location) and organizational leadership that was supportive of creating a team from varied disciplines to share the same role [30].

An exploration of the impacts of COVID-19 on interprofessional education has also just begun. One study, for example, showed that converting a large foundational interprofessional course to an asynchronous learning format still allowed students to meet their IPEC core competencies and understand the importance of engaging in ICP [31]. A case study demonstrated how one interprofessional training program rapidly transformed in-person services to a hybrid telephone-based program with an on-line component, allowing students to continue serving vulnerable communities at the start of the pandemic [32]. In addition to studies that investigated interprofessional education during COVID-19, scholars have called on educators to reimagine interprofessional education to account for the pandemic and its effect on health services. This includes modifying existing interprofessional frameworks to account for on-line interactions; specifying the exact make-up of interprofessional team structures in interprofessional research (e.g., interprofessional team vs. interprofessional collaboration); reconsidering theories underlying interprofessional education and ICP; highlighting critical thinking and creativity, in addition to communication and collaboration, especially in on-line interactions; adapting student performance assessments for the online environment; and addressing issues related interprofessional education and ICP when planning for future pandemics [33]. The existing literature on COVID-19, ICP, and interprofessional education provides a starting point for exploring the experiences of graduate students in early 2020 when COVID-19 led to unprecedented change in the provision of health care services in integrated primary care.

## Methods

### Research design

This qualitative study used focus groups to understand the experiences of 6 interprofessional teams providing mental health services to patients in primary care settings. The teams comprised graduate students in social work, nursing, and rehabilitation counseling and their clinical faculty supervisors. The study was guided by 3 research questions: (a) How did graduate students describe and understand their experiences as part of an ICP training program?; (b) What were the facilitators of and barriers to ICP?; and (c) How was this learning shaped by the emergence of COVID-19?

### Participants and setting

The graduate student sample included 19 individuals between the ages of 23 and 51 (*M* = 34.32; *SD* = 8.17). They identified as female (*n* = 15) and male (*n* = 4) and as non-Hispanic White (*n* = 9), African American or Black (*n* = 7), American Indian/Alaska Native (*n* = 1), Asian (*n* = 1), or Hispanic White (*n* = 1). Participants were enrolled in graduate programs through a large public university in the Northeastern part of the United States: (a) Master of Social Work (MSW) (*n* = 5); (b) post-Bachelor of Science (BSN) in Nursing to Doctor of Nursing (DNP) in Psychiatric Mental Health Nursing (*n* = 6); and (c) post-BSN to DNP Family Nurse Practitioner (*n* = 2), and (d) Masters in Rehabilitation Counseling/clinical mental health (*n* = 6). These participants comprised 6 interprofessional teams providing mental health and psychosocial support services to patients. The 6 teams conducted their educational experience at 6 different agencies. See Table 1 for a detailed description of the agencies, the services they provided, and the effects of in-person practices after the immediate start of COVID-19.

**Table 1:** Agency information and level of integration

Agency	Type of Agency	Graduate Students	Method of Service Delivery	Impact of COVID-19	Level of Integration
Agency 1 (Federally Qualified Health Center [FQHC])	Integrated primary care with behavioral health practitioners (BHPs) delivering mental health services.	Social Work, Psychiatric Nurse Practitioner, Rehabilitation Counseling	In-person till March 19, 2020.	Students continued to see patients remotely.	Agency is fully integrated. PCPs did not always refer patients to students.
Agency 2 (FQHC)	Integrated primary care with BHPs delivering mental health services.	Social Work, Psychiatric Nurse Practitioner, Rehabilitation Counselor	In-person till March 20, 2020.	Students continued to see patients both in-person and remotely.	Agency is fully integrated. Agency supervision sometimes lacking.
Agency 3 (FQHC)	Integrated primary care with BHPs delivering mental health services.	Social Work, Psychiatric Nurse Practitioner, Family Nurse Practitioner	In-person till March 20, 2020.	Students provided a limited number of services remotely.	Agency is fully integrated. But students did not always have opportunities for working directly with PCPs.
Agency 4 (Community clinic)	Integrated primary care with BHPs delivering mental health services.	Social Work, Psychiatric Nurse Practitioner, Rehabilitation Counselor, and Family Nurse Practitioner	In-person till March 20, 2020.	Students had option to provide services remotely. Agency closed in May 2020 due to COVID-19 for summer.	Early stages of integration. Students had many opportunities for providing group therapy but not individual sessions.
Agency 5 (Community clinic)	Integrated primary care agency with drop-in center for homeless population. Offers mental health and psychosocial support. Also serves individuals with HIV.	Social Work, Rehabilitation Counseling, Psychiatric Nurse Practitioner	In-person till March 20, 2020.	Students provided a limited number of services remotely.	Agency is working toward becoming fully integrated. Students had limited opportunities for collaboration.
Agency 6 (Community clinic)	Integrated primary care with behavioral health specialist delivering mental health services.	Social Work, Psychiatric Nurse Practitioner, Rehabilitation Counseling	In-person till March 20, 2019.	Students provided a limited number of services remotely. Clinical supervisor was reassigned, so students had to terminate with patients early.	Agency is fully integrated. Also a training facility for other mental health professionals. Thus, patient flow was limited.

To supplement the perspectives of students and triangulate the data, the sample also included 5 clinical faculty members with at least 4 years of experiencing providing mental health services. The clinical faculty members were from: nursing (*n* = 2), social work (*n* = 1), and rehab counseling/clinical mental health (*n* = 2). Faculty members were not employed by the agencies; they were hired by the project directors of the training program to augment the supervision students received by agency staff.

### Data collection and analysis

Following the conclusion of their year-long specialized interprofessional training program in April 2020, graduate student participants were invited via email to attend a focus group with members of their interprofessional team to reflect on their experiences in the program. The clinical faculty members were also asked via email to take part in a separate focus group to discuss their experiences. All agreed to participate in the groups. In May and June 2020, a videoconferencing platform was used to conduct 6 focus groups with student participants and 1 focus group with clinical faculty participants. Focus groups were used because they are an essential tool for obtaining significant amounts of qualitative data on a variety of experiences in a reasonably short amount of time [34]. They are also helpful or understanding the socio-emotional process connected to these experiences, as they provide direct access to the language that individuals use to discuss their thoughts and feeling about them [35]. The second author (BC) moderated the groups. There was also a process observer in each group (either EA or AZ) to provide additional insight into the investigation and track information that the moderator may have missed [36].

Each focus group consisted of one interprofessional team. All focus groups lasted between 60 and 75 min, were recorded, and professionally transcribed. Student focus group questions included: tell us what it was like for you to be part of an interprofessional team; tell us about the struggles you and your team had in adjusting to your new roles at the primary care agencies, if any; tell us about how your role at the agency evolved over time, if at all; how did the team work together to address patient care needs at your agencies?; tell me about the role of your onsite-clinical supervisor at your agency; and what was it like to deliver interprofessional or discipline-specific patient care during COVID- 19? We reached saturation by the sixth focus group; most of what student participants stated in this group did not offer new data, or data that differed much from the other five groups.

The seventh focus group consisted of 5 clinical faculty members. Focus group questions included: tell us how your initial experiences were with the agencies and students in adjusting to your new role; tell us about your experiences with the agencies that you were assigned to; describe the challenges of working with the system issues and students at the agencies; and describe the support that you received from the agency staff and administration to help the student accomplish their interprofessional roles at the agencies, if at all.

Thematic analysis was used to analyze data. Thematic analysis is a flexible analytic tool used to identify patterns in qualitative data, describe participant experience, and identify the meanings that participants make of this experience [37]. The six phases of thematic analysis include: becoming familiar with the data, generating initial codes, searching for themes, reviewing themes, naming themes, and writing the report [37]. Thus, the fourth author (BG) began the analysis by engaging in repeated reading of the transcripts to ensure he was aware of the depth and breadth of the data. After becoming familiar with the data, he generated a list of codes. After this list was created, the first author (EA) organized the codes and identified preliminary themes. Themes were developed inductively, indicating that we did not rely on existing frameworks or concepts to identify themes and that substantial evidence from the transcripts was required to support each theme [37]. Next, themes were sent to the second (BC) and fifth (PF) authors to review and reach consensus on final themes. Then, the first author produced the report, ensuring that themes formed a cohesive story that balanced text with quotes from students and clinical faculty members [37]. All of the other authors then provided feedback.

### Ethical considerations

We informed students and clinical faculty members that participation was voluntary, that they were being audio-recorded, and that only project team members would have access to the recordings. The institutional review board of Rutgers University approved all study protocols (project number 2020002924).

### Strategies to ensure rigor and trustworthiness

A number of strategies were used to enhance methodological rigor. First, the fourth author (BG), who was not present during the focus groups and not affiliated with the project, conducted open coding. He brought a fresh perspective to the data analytic process. Second, negative case analysis was used; that is, we searched for evidence that disconfirmed particular codes or categories and ultimately the themes. Third, we conducted multiple peer debriefings to review, refute, and refine categories and themes. During these debriefings, we acknowledged our biases and assumptions, making sure to stay as close to the data as possible and not reaching consensus on the final themes until there was agreement. Fourth, all students who participated in focus groups were emailed to review the themes that were identified from the analysis (commonly referred to as member checking); 7 responded to this request. Participants reviewed final themes to ensure they accurately described their experiences [38]. Fifth, the transcript from the focus group with clinical faculty members was triangulated with transcripts from the groups with student participants. Finally, an audit trail was kept to record all decisions and keep track of analytic processes [38].

## Results

Participants reported gaining a beginning level of knowledge and skill when it came to engaging in ICP in the primary care setting, in addition to improving their ability to assess, diagnose, and provide short-term psychosocial interventions to clients dealing with mental health issues. Upon reflection, student participants described their overall interprofessional experience as positive; yet, there were a number of challenges. We identified four themes from the analysis, which are discussed below.

### “You just really have to assert yourself:” encountering difficulties acclimating to the agency and the interprofessional model

Before joining their interprofessional teams at one of the 6 agencies, student participants were required to attend an orientation, participate in online modules that prepared them for integrated care, and engage in training as an interprofessional team by practicing with a simulated patient. Yet, regardless of how prepared students participants were and how immersed the agency was in the integrated model, they described not being ready for what they encountered in the beginning. More specifically, they either did not know what they were supposed to be doing, or did not have enough work to do. Participants reported that they were left feeling frustrated because their services were not being utilized. Thus, they needed to advocate for themselves to ensure that they received adequate clinical training in the last year of their educational program. As Nurse Practitioner Student (NP) 5 expressed: “If we were not assertive, we would have sat around.”

Being able to assert oneself was not always easy, as participants wanted to also be respectful of their role as interns and not seem overly demanding to the agency employees who were busy with their own work. One of the social work student participants who began her experience one semester before joining the interprofessional team in the same agency 4 months later explained:

Social Work (SW) Student 1: …It is funny because I taught a group [on] social skills. We talked a lot about assertiveness and, like, I know to preach it, but I don't know how to role model it … you know, the staff [at the agency] … have heavy caseloads, and they're dealing with … crises and things going on with their clients that I'm like, you just really have to assert yourself into the position or else like you're just going to be sitting in the room at the seat just like talking to random clients …

Student participants described that part of the reason why they had little to do was because PCPs and other staff members were not always willing to engage with them. As SW Student 5 remarked:

I don't know if the doctors fully knew why we were there and what we were doing. Like I think it would have been helpful to have a, you know, like a meeting … to introduce us. To say, … this is what they are there for; this is integrated care.

In addition to finding ways to stay involved in the work flow of the agencies, student participants reported having to acclimate to a new way of providing mental health services. More specifically, they had to move away from practicing psychotherapy or counseling as they would in an outpatient mental health setting to conducting brief sessions in the integrated care setting. As two participants explained:

Rehabilitation Counseling (RC) Student 2: … I'm used to more of behavioral health. So, I'm used to, you know, 45-minute sessions, I'm used to, you know, getting as much information from the client as possible. I think, for me, it was adapting to the integrated care setting and shortened sessions and also working with, you know, two other people on my team. I think that was the biggest adjustment for me.

Nursing Student (NS) 3: … The background I came from … [I] like [to] see [the] patient by myself and taking maybe 45 minutes to assist them and to do the psych [evaluation] … but now having to have everybody coming [in the room to see the patient] … it was like a little [hard] for me at the beginning to really understand …

According to most student participants, acclimating to the agency became more difficult because of the lack of agency administrative supervision, making it more challenging to deal with not having enough to do or not being properly trained on using the electronic medical record. Clinical faculty instructors were hired to advocate for the students while they were at the agencies, and there were times where this helped the adjustment process:

RC Instructor 2: I thought it was great. I just had to advocate a little bit in the beginning to make sure like they knew what my … interns were there for and what their role would be. But that was just like, you know, the first week and then they were like, having them work right away.

However, clinical faculty participants did not always have enough time to deal with these issues. As one clinical instructor participant pointed out:

Nursing Instructor 2: I sometimes thought that I also ran out of time … we would be in the middle of something and the students might get called away for, for some patients, you know, interaction. … so when we were together, sometimes, you know, if we were running out of time, or the students were called away, you know, sometimes I felt that that was a little bit of a challenge.

Although most student participants reported that they needed more guidance in the beginning of their experience, they reported figuring out ways to make things work for them:

NP Student 5: We did a lot of planning. So, we, when we were adjusting to not really having guidance, with a new [electronic medical record], we were able to figure out a way to route list of the schedules for the providers. So, we used that schedule and we would just kind of go through each chart and search for any indication that the patient has a history that needs to be addressed.

### Learning to facilitate team cohesion by engaging in interprofessional communication and learning from others

Despite the difficulties adjusting to the agency, student participants still recognized early on the importance of the interprofessional team and the facilitators of team cohesion as they engaged in interprofessional tasks like huddles and warm handoffs. In fact, they identified interprofessional communication as being a key facilitator of this cohesion. As SW Student 3 stated: “We luckily had a good communication style between [team members]. So, I think that was also just naturally very helpful between us.” Student participants expressed that engaging in effective interprofessional communication allowed them to understand (a) their own limitations when working with patients, and (b) how their fellow team members had a specific knowledge and skill set that was better suited for intervening with a particular patient. As RC Student 2 remarked:

We literally communicated and said … do you feel comfortable doing this? Do you feel more comfortable doing that [given that the patient] has this diagnosis … we just … made sure we were comfortable with whoever we were seeing. And if we weren't, then we brought, you know, an extra person in or the other, you know, all three of us would go in together.

Student participants viewed interprofessional communication as essential for helping them develop mutual respect for one another:

SW Student 2: … I think communication is the biggest piece of it … we will ask each other questions. We weren't afraid to say, when we didn't know something and we also respected the fact that we can have expertise.

In addition to developing their interpersonal communication skills, student participants acknowledged that learning from their team members also facilitated group cohesion. RC Student 2 provided an example, detailing what occurred after one of the PCPs contacted the team to inform them that a patient needed mental health services:

[The] SW Student would go, and I … can also chime in and bring in some counseling skills or you know, even vocational because that's a huge thing too, in rehab counseling. And then DNP Nurse Practitioner Student would go over like medications … so like I think we each brought a little bit of our own, like disciplinary and our own learning. And I think we brought it to the table and it kind of just … meshed really well actually.

SW Student 4 echoed this perspective:

… It gave me a chance to understand different aspects of, you know, working with different professionals … I didn't know much of anything about rehab counseling before …. and getting to learn about, you know, what they do and … how they can be useful to me in the future with other clients that I might have ….

Learning was not limited to student participants just learning from one another. The clinical faculty reported that they also gained insight from their clinical faculty colleagues during the group supervision that they had with the project directors of the program. As two clinical faculty members mentioned:

Nursing Instructor 1: I would agree it was very helpful to just hear what other people were doing at the same time that I was trying to do it. And I felt like I knew what was coming up next. I thought it was really important.

Nursing Instructor 2: I also thought it was really helpful, particularly hearing the experiences that others were having that that was really helpful for me.

### Contending with role confusion and missed opportunities for interprofessional collaboration

As much as student participants were able to effectively engage in interprofessional communication and learn with and from one another, they described barriers to the successful implementation of ICP. One such barrier involved role confusion among agency staff members, who were not always aware what the student participants were supposed to be doing at the agency. This in turn led to competing expectations between agency staff members and student participants, especially in situations where staff members wanted students to be involved in activities that prevented them from engaging with the interprofessional team. For example, NP Student 1, who was enrolled in a family nurse practitioner program, described a situation in which her preceptor wanted her to stay in the office working with the preceptor, rather than co-lead groups with the interprofessional team:

But that's something that [my preceptors] need … to know, that, okay, it's not just about being inside the office, even though it is very important [for] a medical APN to learn [this] stuff, but [I] also need to be [able] to integrate.

In addition to experiencing challenges with agency staff members, student participants had difficulties differentiating the roles and responsibilities of each student team member. In fact, role confusion was common when it came to social work and rehabilitation counseling students at the start of the experience, as their jobs overlapped. Both were both able to provide psychosocial assessments, brief counseling, and refer patients to resources in the community. As one student participant expressed:

SW Student 3: … I can see that [it] might get, you know, awkward if there is a social work student and a rehab counselor student who both want to do everything and want to do one aspect and not the other or so on.

Another student participant expressed a similar sentiment but was also concerned that there could be role confusion with the psychiatric nurse practitioner, too:

RC Student 2: I think a lot of it for me was; we all have mental health backgrounds. And I think so much of it is like, well, we can all kind of do so many similar things. That it was like, well, what am I going to do? What is she going to do? What am I going to do? Like, I think so much of that was my worry.

At the same time, this participant also mentioned that this issue was easy to deal with, especially as time went on: “And then when it actually was put into play, it was just so cohesive. I think between the three of us at least.”

While role confusion was seen as an obstacle that was fairly easy to overcome with effective communication, a more significant barrier to manage was the lack of opportunity to work together as an interprofessional team, even in agencies well known for providing integrated care. As one student participant mentioned:

Interviewer: Did you ever get an opportunity to work together on a patient …?

RC Student 5: Only once … and that's the one time I felt like people are getting together and trying to figure out a way to help the patient on a regular or routine basis … I do not think we ever like sat down and talked about the same patient.

Student participants also mentioned they were concerned that seeing patients as a team might overwhelm them. As a result, they sometimes chose to function independently or to exclude one member of the team when meeting with the patient. As NP Student 8 stated:

I did expect it to be more of a team approach but from when we … had our patient simulation, and we all went in together and we found that that was overwhelming to the patient. I think we took from the experience that we did not need to team up to go in.

It was expected that student concerns related to functioning as an interprofessional team would be addressed in weekly supervision. Although it was evident from the clinical faculty participants that this supervision enhanced students’ clinical skills, its benefits on ICP seemed less apparent. As a result, student participants could sometimes be left with missed opportunities for interprofessional collaboration. RC Instructor 2 remarked:

Sometimes I wish they asked me for more help. They were just so like strong in their own like skills. And, you know, the staff would ask them for advice sometimes, which was nice to see them asking interns for help, and for their input on client cases, but mostly they would ask me for help with counseling skills. So. like motivational interviewing, CBT. So we would do a lot of like, psycho education … [and] helping them with their counseling skills.

### Managing the impacts of COVID-19 on interprofessional learning: uncertainty, frustration, and disappointment

Just as student participants were beginning to understand the dynamics of the agency and patient workflow, their experience was impacted by the COVID-19 crisis in March 2020. As a result, two student teams stopped their placements altogether, while three did phone and telehealth sessions. In one case, only the advanced NP student was providing in-person services, before transitioning to telehealth. As NP Student 5 expressed:

… We were really making progress… like I remember the first Monday, when the pandemic was when things started shutting down. I had like six patients scheduled for me to see and then and I think I only saw one that day because people were not coming.

This participant in particular still had to see patients in-person, even though social work and rehabilitation counseling students were prohibited from providing in-person services. An arrangement such as this one clearly prohibited an interprofessional team approach:

NP Student 5: [It] was difficult because I had to be their roles in a sense, because they were not readily available. And then if I needed anybody, it would be after the fact, you know.

As student participants reflected on the experience, they expressed that much disappointment was caused by not being to terminate with patients as they would have if the pandemic had not occurred. As RC Student 1 stated:

... That was kind of the biggest … disappointment for me … and then not even being able to, like do telehealth or, you know, communicate with your clients. I think that, for me was the biggest struggle because we're so used to, you know, at least in counseling, we're so used to, you know, terminate effectively and with enough time and this and that, and with a pandemic kind of, really didn't give us a chance to do that, unfortunately.

At the same time, student participants expressed that they were dealing with their own personal issues related to the pandemic, while trying to manage their educational and field work experience. This led to some questioning whether they should be helping patients during this time. For instance, SW Student 5 explained:

It was stressful because we were I felt like I was also dealing with my own anxiety during the whole thing and trying to talk to but I, it was for me it was a little bit stressful, but as I got more into it, it became it was helpful and I thought like patients appreciated it and I was able to help patients. So, in that aspect, it was good.

The effects of the pandemic could be even harder to manage when student participants were providing childcare. As RC Student 3 expressed: “… With all the stress and especially with my daughter being all the time like on top of me, I felt … I was not productive in helping other people.” Given the stress and anxiety that was coming up for students during the pandemic, clinical instructor participants expressed that they had to take on a counseling role:

RC Instructor 2: I mean, one student couldn't graduate on time, so they're like a lot of tears. One lost a family member during the Coronavirus. So just being there for them really just showing empathy and you know, extending those hours just text me or call me whenever you need me.

Similar to Counseling Instructor 2, others felt the need to be flexible since the student participants were dealing with high levels of stress at the beginning of the COVID-19 pandemic. There were situations when the clinical instructors did not even know how to respond to students, given how uncertain things were are the time. Thus, much of the time was spent listening and providing them with an opportunity to share their feelings and concerns. As RC Instructor 1 indicated:

I definitely had a lot of one-to-one conversations with students … a lot of just hearing how they were doing and how they are feeling. And, you know, not being able to provide them with any answers as to what was going to happen. That often came up, people would often come to me looking for answers … so, you know, was really mostly about processing what they were going through. And there was a lot of emotions during that time. Yeah. So that is mostly what we did. We did a lot of zoom meetings. And, like the other [participant] said, being flexible.

All opportunities for learning were not lost during the pandemic, however. Four student participant teams ended up using telehealth to see patients, enabling them to provide continuity of care and continue supporting one another. As RC Student 6 commented, “[we leaned] on each other as coworkers more because, you know, no one had experienced anything like this before.” The use of telehealth also allowed these student participants to keep learning from one another. It also helped them to feel less alone when working with patients they were worried about:

NP Student 8: …. I shared [my concern] with the MSW SW Student, RC Student, and [my clinical supervisor] gave me great feedback on how good of a job I actually did, even though I was kind of beating myself up because it was telehealth … so, I did the best that I could.

## Discussion

The aim of this study was to understand the experience of healthcare students working in interprofessional teams delivering behavioral health services in primary care settings. The themes underscore the facilitators of and barriers to their experience and describe what it was like to practice in integrated primary care at the start of COVID-19. Overall, student participants had a positive experience; however, they also encountered challenges that sometimes impacted their abilities to engage in ICP. First, student participants struggled when they first arrived at their agencies. While it was expected that they would have some issues in the beginning, the extent of these issues was surprising. Student participants reported that they did not have enough work to do and did not know exactly what they should be doing. We assumed that this would occur in agencies that did not use the integrated care model but were surprised this also took place in agencies already using this model. The lack of clear direction impacted the beginning phase of entry for student participants, making it harder to attain the ICEP competencies. Similar to previous studies [39, 40], student participants also had difficulty shifting from typical 45-min psychotherapy sessions to brief encounters required to practice effectively in the integrated care setting. Challenges were exacerbated by clinical faculty members being unable to fully assist student participants with integrating into the workflow of the agency. The difficulty of providing interprofessional services in agencies with an established work flow has previously been discussed [2], suggesting that what student participants faced in their agencies was not entirely uncommon. Our findings also align with previous research demonstrating there are sometimes discrepancies between the core goals and functions of the interprofessional team and what actually occurs in real-world behavioral health practice [41, 42].

Ultimately, student participants were able to provide high-quality integrated services as their experience continued. Student participants highlighted the importance of clear communication and using their experience to learn from one another, which was consistent with prior research [43]. It was apparent from focus group transcripts that students were able to meet the competency of interprofessional communication. Indeed, student participants benefited from this type of communication, as it allowed them to rely on team members to increase their understanding of a specific patient issue or to defer to teams member who had more experience than them. In the integrated care setting, communication has often been considered critical to developing the interprofessional team [14], and this was no different for student participants in the current study. Findings indicate that student participants understood the importance of engaging in interprofessional communication in order to provide effective mental health services and psychosocial support, emphasizing their professional growth in this area. Professional growth, in fact, has often been highlighted as a significant outcome of providing interprofessional care [2]. Should these student participants continue to provide services in an integrated care setting, findings indicate it is likely they will be able to work cohesively as a team, understand the importance of effective interprofessional communication, and gain additional perspectives to guide services.

While the competency related to interprofessional communication was easy to meet, student participants reported having trouble understanding the role of each team member in the beginning. This was especially the case for the social work and rehabilitation counseling students. Their roles overlapped considerably; thus, they were understandably concerned that each would get pushed aside. However, each student participant was able to gain insight into their particular roles and how these roles overlapped in the provision of team-based care, which aligns with competency two of the framework. Despite this concern, student participants expressed they missed opportunities to work as interprofessional team. They were either asked by their agencies to function independently, even by the agencies that were supposed to be providing integrated care. This was contradictory to the IPEC framework and led to a gap in training. And when the chance to function as an interprofessional team did arise, the student participants sometimes worried that seeing patients all together might overwhelm them and thus they functioned independently, which could be attributed to the lack of experience in ICP.

Overshadowing the student participant experience was the need to provide interprofessional services at the start of the COVID-19 pandemic. COVID-19 has been shown to affect service delivery, shifting the delivery of in-person behavioral health and primary care services to telephone or through virtual platforms [32, 44, 45], and it was no different for student participants in this study. The impact of COVID-19 on their experience was significant. They had to stop working together as a team right after building cohesion, leaving a few students providing in-person services or telephone or virtual services. Nevertheless, they were able build on the communication skills they developed earlier in their training to continue providing services and finish out their experiences. Many expressed disappointment at being unable to witness the full potential of delivering integrated services and to terminate with patients in ways that were sensitive to their needs, especially those with more serious psychiatric disorders.

The shift to telehealth during the pandemic presented unique communication challenges that came with the virtual care that student participants had to provide due to COVID-19. Additionally, student participants who did provide telephonic or online counseling grappled with caring for patients while taking care of themselves and their families. Clinical faculty participants played an important role in helping student participants deal with the obstacles that impacted patient service delivery and the functioning of the interprofessional team. This meant that clinical faculty participants also had to recognize and sometimes even help address the personal issues that arose for students because of the COVID-19 pandemic. Given the extraordinary nature of the pandemic, providing this support was deemed necessary for student participants to complete fieldwork without increasing their stress levels.

### Implications for Interprofessional collaborative practice

Our findings align with Reiter et al. [42] who indicate that discrepancies sometimes exist between the core goals and functions of the interprofessional team and what actually occurs in real-world behavioral health practice. Thus, findings have implications for maximizing the interprofessional learning that social work, nursing, and rehabilitation counseling students engage in. Table 2 connects each theme to its interprofessional practice implications. We also discuss implications for interprofessional practice below.

**Table 2:** Themes and their Implications for Training Healthcare Students to Provide Team-Based Behavioral Health Care Services in Primary Care Settings

Themes	Implications for Educators, Clinical Faculty, and Agency Supervisors Working to Improve Interprofessional Learning at Clinical Agencies
Encountering difficulties acclimating to the agency and interprofessional model	• Educate primary care providers (PCPs) about the purpose of the interprofessional team (i.e., to assist patients struggling with mental health and psychosocial problems) and the benefits of interprofessional collaborative practice (ICP). • Provide student opportunities for meeting PCPs and other agency staff members who can serve as referral sources. • Delineate student responsibilities and assignments from the outset, including whether students will have access to the electronic medical record. • Advocate for students to agency personnel, so they have patient contacts on a daily basis and engage in team-based learning regularly. • Bolster student confidence in their ability to transition from conducting typical 45-min psychotherapy sessions to brief, targeted counseling sessions.
Learning to facilitate team cohesion by engaging in interprofessional communication and learning from others	• Emphasize the importance of having student teams engage in warm handoffs and huddles even in agencies where full integration does not exist. • Model effective interprofessional communication skills for students regardless of the agency’s level of integration. • Highlight the role of interpersonal communication in facilitating team cohesion. • Provide opportunities for educators, clinical faculty, and agency supervisors to process their experiences with student teams as a method for improving interprofessional learning as a whole.
Contending with role confusion and missed opportunities for interprofessional collaboration	• Work closely with agency supervisors to orient students, so they clearly understand their professional role within the agency. • Normalize role confusion and help students understand how their roles are similar and different. • Train student teams members to recognize and address the communication barriers preventing the team from functioning properly. • Encourage students to engage in interprofessional communication and integrated treatment planning even in situations where only one student sees the patient. • Conduct weekly meetings with students to identify team strengths and solutions for addressing communication barriers.
Managing the impacts of COVID-19 on interprofessional learning: Uncertainty, frustration and disappointment	• Help students anticipate the impact of a potential public health emergency on patient care and team-based patient care. • Develop a contingency plan that allows for students to quickly transition from providing sessions in-person to virtually. • Devote significant amount of time to teaching students how to incorporate ICP strategies in virtual environments. • Ensure there is a self-care or wellness component incorporated into student training that prepares them to manage the emotional effects of practicing during a public health emergency.

First, academic and clinical faculty members should work much more closely with agency supervisors to orient students to ensure they clearly understand their role within the agency and that their work assignments are delineated from the outset, including whether they will have access to the electronic medical record. Second, agency staff should educate PCPs about the purpose of the interprofessional team and the benefits of ICP, so that students are able to provide assistance for patients struggling with mental health and psychosocial problems. Opportunities should be set up for students to meet PCPs and other agency staff members who can serve as referral sources.

While it is hard to deny the complex realities of the agency environment, students should not be pulled from their interprofessional teams to provide behavioral health services independently, even in agencies where full integration does not exist. This is counterintuitive to the tenets of interprofessional care and may subsequently create role strain for students. Similarly, supervisors working in agencies that are not fully integrated should help students understand which situations constitute the entire interprofessional team engaging the patient together, and when one team member should take responsibility for engaging the patient. When it is determined that only member of the team will see the patient, supervisors should point out that the team should still work to together to conceptualize the case and create an integrated care plan. The integration of services may help reduce a delay in treatment and improve service delivery, just a few of the many advantages of working within a team [1, 46]. Supervisors should model effective communication skills for students regardless of the agency’s level of integration, so these skills are used within the interprofessional team [43]. Team members should also be trained to recognize and address the communication barriers that prevent the team from functioning properly. It will also be important to increase opportunities for team members to learn from one another and feel comfortable deferring to another team member who may the particular expertise to treat specific patient problems. As participants in this study expressed, and has been emphasized in previous literature on COVID and interprofessional practice and education [28, 31–33], educators should also focus on teaching ICP strategies in an on-line environment. This would enable students to provide much needed virtual services in ways that effectively meets the needs of patients who require routine behavioral health services but cannot attend in-person sessions because of a public health or other type of emergency. COVID-19 has transformed the way health services are delivered, especially particular behavioral health. And the use of telemedicine and telepsychiatry provides increased flexibility and access for populations who in the past were not able access such services. Thus, students should be educated about how to manage patients virtually in order to deliver evidence-based services that meet the myriad needs of individuals seeking health services in primary care settings.

### Limitations

There are noteworthy limitations to this study. First, focus groups were conducted with only one cohort of the training program. Including students from other cohorts would have allowed for even richer data. For example, future research would benefit from including students from multiple cohorts, as this would allow for a comprehensive look at how their experiences vary based on being in the field when the COVID-19 pandemic started and entering the field in the middle or end of the pandemic. Second, only one method of data collection was used. Although focus groups enable researchers to obtain large amounts of data in short periods of time [27], individuals might not always feel comfortable sharing certain information in front of others. The use of individual interviews would have also allowed for additional data triangulation. Third, focus groups were conducted by the project director of the grant, an individual who student participants had been in contact with for their duration in the program. Thus, it is possible that they might not have always felt comfortable discussing their perspectives; however, it is important to mention that participants seemed to appreciate the opportunity to share information and empowered to discuss the challenges they encountered. Finally, it would have also been important to explore the perspectives of agency staff and supervisors. This could have provided more context for understanding the challenges that student participants experienced. In fact, future research should explore the perspectives of agency staff, supervisors, and primary care physicians to identify barriers to implementing a student interprofessional team, as well as solutions for addressing these barriers.

## Conclusion

Despite the study’s limitations, it highlights students’ experiences providing behavioral health services in primary care settings; another building block to support the overall implementation of this much-needed care, especially as the COVID-19 pandemic continues. The study also illuminated important implications directly from the experiences of interprofessional team members that call for academic faculty to work much more closely with clinical and agency supervisors to orient students to ensure they clearly understand their role within the agency and on the interprofessional team, particularly when providing integrated care on-the-ground *and* in the virtual environment.

## Data Availability

The first and second authors (EA and BC) own the data. The datasets generated during and/or analyzed during the current study are not publicly available due to concerns regarding participant confidentiality; however, data are available from the authors upon reasonable request.
